# Photon-Counting Computed Tomography Angiography of Carotid Arteries: A Topical Narrative Review with Case Examples

**DOI:** 10.3390/diagnostics14182012

**Published:** 2024-09-11

**Authors:** Antonella Meloni, Riccardo Cau, Luca Saba, Vincenzo Positano, Carmelo De Gori, Mariaelena Occhipinti, Simona Celi, Eduardo Bossone, Jacopo Bertacchi, Bruna Punzo, Cesare Mantini, Carlo Cavaliere, Erica Maffei, Filippo Cademartiri

**Affiliations:** 1 Bioengineering Unit, Fondazione G. Monasterio CNR-Regione Toscana, 56124 Pisa, Italy; antonella.meloni@ftgm.it (A.M.); positano@ftgm.it (V.P.); 2 Department of Radiology, Fondazione G. Monasterio CNR-Regione Toscana, 56124 Pisa, Italy; carmelo.degori@ftgm.it (C.D.G.); mocchipinti@ftgm.it (M.O.); 3 Department of Radiology, University Hospital of Cagliari, 09042 Cagliari, Italy; riccardocau00@gmail.com (R.C.); lucasabamd@gmail.com (L.S.); 4 BioCardioLab, Fondazione G. Monasterio CNR-Regione Toscana, 54100 Massa, Italy; simona.celi@ftgm.it; 5 Department of Cardiology, Antonio Cardarelli Hospital, 80131 Naples, Italy; ebossone@hotmail.com; 6 Leeds General Infirmary, Leeds Teaching Hospitals NHS Trust, Leeds LS1 3EX, UK; jacopo.bertacchi1@nhs.net; 7 Department of Radiology, Istituto di Ricerca e Cura a Carattere Scientifico SYNLAB SDN, 80131 Naples, Italy; bruna.punzo@synlab.it (B.P.); carlo.cavaliere@synlab.it (C.C.); ericamaffei@gmail.com (E.M.); 8 Department of Radiology, “G. D’Annunzio” University, 66100 Chieti, Italy; cesare.mantini@gmail.com

**Keywords:** photon-counting computed tomography, carotid arteries, photon-counting detectors

## Abstract

Photon counting computed tomography (PCCT) represents a paradigm shift from conventional CT imaging, propelled by a new generation of X-ray detectors capable of counting individual photons and measuring their energy. The first part of this narrative review is focused on the technical aspects of PCCT and describes its key advancements and benefits compared to conventional CT but also its limitations. By synthesizing the existing literature, the second part of the review seeks to elucidate the potential of PCCT as a valuable tool for assessing carotid artery disease. Thanks to the enhanced spatial resolution and image quality, PCCT allows for an accurate evaluation of carotid luminal stenosis. With its ability to finely discriminate between different tissue types, PCCT allows for detailed characterization of plaque morphology and composition, which is crucial for assessing plaque vulnerability and the risk of cerebrovascular events.

## 1. Introduction

Acute ischemic stroke presents a considerable worldwide challenge, causing significant morbidity and mortality [1]. It accounts for approximately 5% of the world’s disability-adjusted life-years, and it is responsible for more than 10% of global deaths [2]. To improve patient care and optimize outcomes, it is crucial to differentiate between the various causes of stroke [3,4]. The most widely used system in differentiating stroke etiologies is the Trial of Org 10172 in Acute Stroke Treatment (TOAST). This classification system discriminates five subtypes of stroke etiologies, namely, large-artery atherosclerosis, cardioembolism, small-vessel occlusion, stroke of other determined etiology, and stroke of undetermined etiology [4]. In this scenario, a detailed evaluation of carotid artery disease is essential for effectively stratifying risk and managing individuals with cerebrovascular ischemia caused by carotid atherosclerotic disease [5]. If significant carotid atherosclerosis diseases are detected as the cause of symptoms, these patients may be eligible for a carotid intervention aimed at preventing a subsequent stroke [6].

Traditionally, the degree of carotid luminal stenosis has been pivotal in assessing the risk of ischemic stroke and deciding on the potential need for surgical intervention based on prior randomized trials, namely the European Carotid Surgery Trial (ECST) and the North American Symptomatic Carotid Endarterectomy Trial (NASCET) [7,8]. However, recent evidence underscores that factors beyond the degree of luminal stenosis may significantly influence the clinical manifestations of atherosclerotic plaques. In particular, certain imaging characteristics of plaque composition and morphology, indicating vulnerability, could act as independent risk factors in initiating cerebrovascular events, regardless of the degree of stenosis [9,10]. The main imaging biomarkers related to carotid plaque vulnerability, as demonstrated in histological studies, encompass plaque thickness, intraplaque hemorrhage (IPH), plaque ulceration, thin or ruptured fibrous cap (FC), the existence of a lipid-rich necrotic core (LRNC), calcification type, inflammation, and neovascularization [10,11,12,13,14,15,16].

The Carotid Plaque-RADS stroke risk classification system has been recently introduced. This newly developed cross-modality scoring system is tailored for comprehensive reporting on carotid plaque using non-invasive imaging modalities. It considers both carotid luminal stenosis and specific imaging features indicating plaque vulnerability [9].

Among non-invasive imaging modalities in clinical practice, computed tomography (CT) is recognized as a powerful non-invasive tool for evaluating the atherosclerotic process. It allows for a thorough assessment of both luminal stenosis and plaque composition and morphology [17,18]. CT enables the examination of plaque surfaces and provides valuable insights into plaque composition through analysis of Hounsfield unit (HU) densities [17,18]. However, the diagnostic accuracy of conventional CT is hindered by restricted spatial resolution and the presence of severely calcified plaques. Carotid calcifications can result in an overestimation of luminal narrowing because dense calcifications may influence the density of neighboring voxels, leading to an exaggerated depiction of the calcified lesion. Consequently, this overestimation can affect the accuracy of vessel stenosis assessment [19]. Additionally, CT is limited in the evaluation of features of plaque vulnerability, such as FC thickness, due to limited spatial resolution and artifacts like the blue edge and halo effect, and the presence of plaque subcomponents, due to the overlap of HU values between LRNC, fibrous tissue, and IPH [20,21].

In recent years, photon-counting CT (PCCT) has emerged as a promising next-generation CT technology, enabled by cutting-edge advancements in X-ray detector technology. PCCT offers several distinct advantages over conventional CT imaging and addresses some inherent limitations [22].

This narrative review provides an overview of the principles, technical advancements, strengths, and limitations of PCCT technology, and, by synthesizing the existing literature and highlighting key findings from preclinical and clinical studies, it seeks to elucidate the potential of PCCT as a valuable tool for the assessment of carotid artery disease.

## 2. Comparison between Energy-Integrating and Photon-Counting Detectors

Conventional CT scanners utilize energy-integrating detectors (EIDs) containing scintillator elements and reflective layers known as septa [22,23,24]. The scintillators convert the incoming X-ray photons into visible light photons. These light photons are then captured by a photodiode array composed of semiconducting material, generating an electrical signal proportional to the total energy absorbed. Afterwards, this electrical signal undergoes amplification and conversion into a digital format for the image reconstruction process. Since the detector integrates the energy from all incident photons within a specified timeframe, individual X-ray photon energy details are lost [24,25]. The septa channels filter the produced visible light, reducing the crosstalk between adjacent scintillator pixels but resulting in inactive areas on the detector surface. Since their size cannot be reduced under a certain level for mechanical and optical reasons, the reflective septa reduce the fill factor and the dose efficiency of conventional CT [23,24].

Photon-counting detectors (PCDs) enable the direct conversion of X-ray photons into electrical signals [26,27,28]. At their core, PCDs consist of a semiconductor layer, typically composed of materials like cadmium telluride (CdTe), cadmium zinc telluride (CdZnTe), or silicon. The semiconductor layer is sandwiched between a large-area cathode electrode located on the upper side and pixelated anode electrodes positioned on the lower side. When a high voltage, usually ranging between 800 and 1000 volts, is applied across the cathode and the individual anodes, it creates a strong electric field within the semiconductor layer. The semiconductor layer absorbs the incident X-ray photons, generating electron-hole pairs. Under the influence of the electric field, these electron-hole pairs separate and migrate towards the anode electrodes. As electrons reach the anodes, they induce short current pulses. An electronic pulse shaping circuitry is employed to convert these current pulses into voltage pulses. The height of these voltage pulses is directly proportional to the incident X-ray photon’s energy, enabling PCDs to provide accurate energy information for each detected photon. The output signal from the PCD, consisting of voltage pulses, undergoes further processing using electronic comparators and counters [24,25]. The heights of the voltage pulses can be compared with a predefined voltage corresponding to a specific photon energy level (energy threshold) [29]. The photon count is incremented by one for each detected photon that surpasses the threshold, allowing PCDs to effectively measure the intensity of X-ray photons at a specific energy level. By employing multiple energy thresholds, PCDs conduct a comparative analysis of all pulses, thereby categorizing incident photons into distinct energy groups or bins. This process enables the differentiation of X-ray photons based on their energy levels, with the number of energy bins typically ranging from 2 to 8. Setting the lower threshold higher than the electronic noise level is a common practice in PCDs to ensure effective elimination or suppression of noise in the final signal. The other thresholds can be uniformly spaced, simplifying the implementation and calibration of the detector system, or strategically configured to fine-tune the energy discrimination capabilities to match specific imaging objectives, thereby enhancing imaging quality and clinical utility [30].

## 3. Strengths of PCDs

This section briefly describes the advantages offered by PCCT systems over conventional EID-CT modalities.

### 3.1. Enhanced Spatial Resolution

Spatial resolution is a critical factor in CT imaging as it directly impacts the ability to distinguish small or fine details accurately. In clinical practice, high spatial resolution is particularly important for imaging tasks that require visualizing small anatomical structures or subtle abnormalities, such as in neurological imaging. The spatial resolution in a CT image relies on multiple factors, including the size of the X-ray focal spot, the size and number of detector elements, and the reconstruction algorithm used to create the image [31]. Smaller detector elements or pixels can capture finer details of the scanned object, while advanced reconstruction techniques can further enhance image sharpness.

Clinical EIDs typically feature pixel sizes of approximately 0.4–0.6 mm at the isocenter [32]. The necessity for highly reflective layers challenges reducing the size of detector elements. While thinner septa are desirable to accommodate smaller pixels, overly thin septa can exacerbate the occurrence of photon crosstalk. Moreover, diminishing the size of detector pixels inherently reduces the overall sensitive area of the detector to X-rays. This reduction translates into decreased geometric dose efficiency, potentially compromising image quality and diagnostic efficacy [27].

In PCDs, the absence of reflectors or dead areas between pixels maximizes the effective utilization of the detector surface. PCDs can implement smaller pixel sizes, ranging from 0.15 to 0.225 mm at the isocenter while maintaining optimal geometric efficiency [33,34,35,36].

### 3.2. Improved Noise Characteristics

The measured signal in EIDs consists of the desired signal from X-ray photons and the background noise from the detector system. This electronic noise can arise from various sources, including thermal fluctuations, electronic components, and environmental factors.

In PCCT, the threshold-based photon-counting mechanism helps mitigate the effects of electronic noise. By setting the lower threshold above the electronic noise level (approximately 25 keV), PCDs selectively count only those photons whose energy exceeds the threshold, effectively filtering out noise-induced signals below this level [37].

Thanks to the ability to eliminate the electronic noise, enhancing image quality and diagnostic accuracy, PCDs are inherently more dose efficient than conventional EIDs. Therefore, PCDs are invaluable tools in low-dose CT imaging scenarios, where maintaining image quality while reducing radiation exposure is crucial, and in the scanning of patients with high body mass or larger body habitus, for whom the increased attenuation of X-rays can increase the levels of noise [38,39].

### 3.3. Improved Contrast

In conventional EIDs, photons are commonly weighted according to their energy levels, with higher-energy photons carrying greater influence on the overall signal compared to their lower-energy counterparts. The underweighting of low-energy photons, which contain valuable information about material contrast, can detrimentally impact the contrast-to-noise ratio (CNR), a critical metric for image quality assessment [27,40]. Furthermore, the non-uniform weighting of photons introduces variability in the signal output, resulting in increased variance relative to the mean signal value. In turn, this elevated variance reduces the signal-to-noise ratio (SNR) as the noise level becomes more pronounced relative to the signal intensity. This phenomenon is intimately linked to the Swank factor, which characterizes the variability in detection efficiency across different photon energies within the detector material [41].

In PCDs, all photons are treated equally, irrespective of their energy level, by adopting a one-photon, one-count approach. By assigning equal weight to all photons, PCDs inherently prioritize the detection of low-energy photons, which can lead to higher contrast, particularly for materials with low X-ray attenuation [42,43,44,45]. An additional benefit of PCDs lies in their flexibility, as the weighting scheme can be customized to optimize the CNR for specific materials or imaging tasks, allowing for enhanced image quality and diagnostic accuracy in diverse clinical applications [27,46,47].

By offering a more uniform response to photons of varying energies, PCDs ensure consistent signal detection and mitigate the inherent limitations associated with the Swank factor.

### 3.4. Enhanced Capabilities of Spectral Imaging and Material Characterization

Conventional CT primarily offers anatomical and morphological evaluation of organs and tissues, relying on qualitative assessment based on the attenuation of X-rays at a single energy level chosen during acquisition. Spectral CT adds a new dimension to the images by acquiring data at multiple energy levels [48,49]. The fundamental principle underlying spectral CT is the energy dependence of X-ray attenuation coefficients for different materials.

By comparing attenuation levels derived from high and low energy levels, spectral CT algorithms calculate the contributions of the photoelectric effect and Compton scattering, facilitating the separation of tissues with similar attenuation at any single energy level. Tissue separation is commonly named material decomposition and is the basis for spectral CT imaging. Basis material decomposition involves defining a set of basis materials with known attenuation properties and determining their relative contributions to the measured attenuation at each energy level. By solving a system of equations, the concentrations of these basis materials can be estimated, providing quantitative information about tissue composition. The number of materials or bases corresponds directly to the amount of spectral data gathered during imaging (N spectral data points = N bases) [50]. The imposition of mass or volume conservation constraints allows the expansion of the spectral data to N + 1 levels, but this can potentially affect the accuracy of the material decomposition process [51]. Various visualization techniques can be employed to extract comprehensive information from spectral CT data. Material-specific images highlight the distribution of specific materials within the scan, providing insights into tissue composition and aiding in detecting and characterizing lesions. Virtual non-contrast (VNC) images are generated by digitally removing the contrast agent signal from contrast-enhanced images, providing a baseline anatomical reference without the need for a separate pre-contrast scan, reducing radiation dose and scan time images [52,53]. Iodine maps highlight the distribution and concentration of iodine within the scanned area, aiding in assessing perfusion and vascular abnormalities [54,55]. Energy-selective images, such as virtual monochromatic images (VMIs), simulate scans acquired at a single energy level, allowing for optimization of contrast and noise characteristics [56,57]. Low-keV images increase contrast and lesion visibility, while high-keV images mitigate beam-hardening artifacts [56,57,58]. Intermediate energy images (60–75 keV) strike a balance between contrast enhancement and noise reduction and are ideal for assessing soft tissues [59].

Dual-energy CT (DECT) using EIDs collects data in two energy regimes and can, therefore, distinguish up to three types of materials (under the assumption of volume or mass conservation) [50]. Moreover, DECT is prone to spectral overlap, which compromises the accuracy of material decomposition. PCDs inherently overcome these limitations. The capability to differentiate photons with different energies allows PCDs to characterize the composition of each voxel as a combination of three or more basis materials and to perform multi-energy spectral CT with perfect temporal and spatial registrations and without spectral overlap [60]. Increasing the number of energy regimes results in superior material-specific or weighted images [61] and VNC images [56] and enables novel approaches for material decomposition in spectral CT, including techniques like K-edge imaging.

K-edge imaging involves finely tuning the energy bin boundaries to align closely with the K-edge energy of specific elements, defined as the binding energy between the inner electronic layer and the atom [62]. This configuration allows for the precise identification and quantification of specific contrast agents within the scanned volume based on their distinct K-edges, hence offering a unique opportunity to utilize various contrast agents beyond iodine, including gadolinium, gold, platinum, silver, ytterbium, and bismuth [63,64,65,66]. K-edge imaging is promising for various applications, such as multi-contrast agents and molecular imaging. Multi-material imaging allows for the precise assessment of the distribution of different contrast agents administered simultaneously or the visualization of various contrast agents with distinct distribution properties [24,40]. Molecular imaging enables the real-time visualization of cellular functions and corresponding molecular processes. It plays a crucial role in various diseases diagnosis, predicting their prognosis, and guiding the selection of appropriate treatments [67,68,69]. Molecular imaging requires innovative contrast agents with high affinity and specificity, such as nanoparticles targeted to particular cells or enzymes [68,69,70,71].

### 3.5. Reduced Artifacts

Artifacts are common in clinical CT imaging and can potentially obscure anatomical details and simulate or obscure true pathology, compromising diagnostic accuracy.

Beam-hardening artifacts occur due to the differential attenuation of X-ray photons passing through an object containing materials with varying densities. This phenomenon results in a shift in the energy spectrum of the X-ray beam towards higher energies, leading to increased penetration of low-density tissues and increased absorption by high-density tissues [72]. The consequence of beam hardening is the production of streaking or shading artifacts in the reconstructed CT images, particularly around high-density structures like bone [73].

PCDs offer a unique advantage in reducing beam-hardening artifacts through their constant weighting approach [74,75]. The achieved normalization of attenuation measurements across different energy levels reduces the variability in photon attenuation, resulting in clearer and more accurate CT images.

Furthermore, PCDs can utilize high-energy thresholds as a filtering mechanism to selectively exclude photons that are more susceptible to causing beam-hardening artifacts [38,76].

Calcium blooming and metal artifacts are complex phenomena influenced by multiple factors, including volume averaging, motion, and beam hardening [77]. In PCDs, the reduction of these artifacts is notably pronounced. This improvement stems from the enhanced spatial resolution, which minimizes partial volume effects, and the improved material decomposition, accurately distinguishing high-density materials like metals from adjacent soft tissues [78].

Motion artifacts, often occurring when patients inadvertently move during the scan, particularly in longer examinations, can significantly degrade the quality of CT images, leading to blurring or distortion of anatomical structures. Compared with conventional EID-CT systems, the faster acquisition times of PCCT systems allow for quicker image acquisition, decreasing the likelihood of motion-related image degradation.

## 4. Limitations of PCCT

PCCT faces technical, clinical, and economic challenges that must be addressed to realize its full benefits. Ongoing research and development efforts to overcome these limitations are essential for advancing the field of PCCT and unlocking its transformative capabilities in diagnostic imaging.

### 4.1. Technical Challenges

The main technical challenges of PCCT are pulse pile-up, charge sharing, and K-fluorescence escape [23,79].

Pulse pile-up arises when two or more X-ray photons are detected within the same time window and are recorded either as a single pulse with energy equal to the sum of the original pulses or as overlapping pulses with incorrect energy levels. Consequently, individual photons’ accurate counting and energy measurement are compromised [80,81]. Smaller pixels could be used to address the counting rate problem. Anyway, pulse pile-up is usually not a significant issue at X-ray flux rates relevant to medical imaging [80].

Charge sharing is caused by the interaction of an incident X-ray photon near the boundary of two adjacent pixels. Instead of being localized to just one pixel, the charge generated by the photon is distributed across neighboring pixels. This results in the detection of lower-energy events in these adjacent pixels, leading to image artifacts and a reduction in spatial resolution [62,82,83]. Advanced detector designs with optimized pixel configurations and improved charge transport properties are employed to mitigate the effects of charge sharing. Additionally, sophisticated signal processing techniques are utilized to compensate for these phenomena, enhancing energy measurement accuracy in photon-counting detectors.

K fluorescence escape occurs when secondary X-ray photons generated within a pixel propagate to adjacent pixels, leading to a false increase in detected signals [37]. This effect imposes a lower limit on the feasible pixel size in PCCT applications.

In developing PCCT, optimizing detector pixel size is a key objective, aiming at achieving the necessary energy separation while maintaining the highest possible spatial resolution and effectively managing pile-up effects [84].

### 4.2. Clinical Considerations

#### 4.2.1. Alternative Contrast Agents

The alternative contrast agents offer promising potential for addressing specific clinical needs and pushing the boundaries of imaging towards enhanced diagnostic precision and tailored patient care. However, integrating alternative contrast agents in PCCT requires addressing challenges such as biocompatibility, tissue specificity, imaging artifacts, and cost. Rigorous preclinical and clinical evaluations are necessary to ensure the safety and efficacy of alternative contrast agents. At the same time, optimizing imaging protocols and reconstruction algorithms is essential to mitigate imaging artifacts and enhance diagnostic accuracy.

Importantly, many alternative contrast agents are still experimental or investigational and have not received regulatory approval for clinical use. Obtaining regulatory clearance can be a lengthy and resource-intensive process, delaying the availability of contrast agents for routine clinical imaging.

#### 4.2.2. Clinical Translation and Validation

Despite promising preclinical research, the literature delineating the clinical applications of PCCT is still limited, and most clinical studies are characterized by small sample sizes. Moreover, there is a lack of standardized acquisition protocols across institutions, resulting in significant variability in image quality and diagnostic performance. Large-scale studies encompassing diverse patient cohorts and implementing standardized acquisition settings are needed to enable robust comparisons and generalizability of findings and to fully elucidate the diagnostic accuracy, efficacy, and clinical relevance of PCCT.

#### 4.2.3. Data Handling and Analysis

In general, conventional post-processing software can provide analytic capabilities of basic information. However, there may be issues with ultra-high resolution (matrix 1024 × 1024 and large 0.2 mm slice thickness datasets) and spectral datasets for quantitative purposes that are proprietary of the vendor. Each standard exam can be 5–10 GB when all high resolution and spectral capabilities are exploited. This demands substantial computational power and also places a significant burden on radiologists, who must analyze the increasing volume of images and generate the corresponding reports. To prevent any loss of information, additional technical and human resources may be required, which could be challenging, particularly in an era of diminishing resources. Image transfer can be limited by non-up-to-date network infrastructure.

### 4.3. Economic Considerations

The high cost associated with developing and manufacturing PCDS represents one of the major obstacles to the widespread clinical adoption of PCCT [85]. Conversely, the cost discrepancy between PC and conventional CT scanners may diminish over time as more healthcare organizations adopt this emerging technology.

## 5. Scanning and Reconstruction Protocols

The current clinical photon-counting CT systems feature three main scanning modes: ultra-high resolution (UHR), multi-energy, and dual-source modes. The UHR mode offers the highest spatial resolution. Multi-energy mode enables the production of virtual monochromatic images at standard scanning speeds. The dual-source mode is designed for extremely fast scans. The selection of the imaging mode should align with the specific objectives of the examination. The multi-energy mode is commonly used for neurological scans due to its versatility in spectral imaging.

The raw data obtained from the PCCT system is distinct from that gathered by conventional CT systems. As a result, the image reconstruction process for PCCT data must be handled differently. The clinically adopted reconstruction method is known as Quantum Iterative Reconstruction (QIR), developed by Siemens Healthineers (Erlangen, Germany). The PCCT scanner is equipped with a wide variety of reconstruction kernels, and both the kernel and QIR algorithm settings need to be considered together. Typical acquisition and reconstruction parameters for a carotid angiography using PCCT are reported in the Figure legends.

## 6. PCCT in Carotid Arteries Assessment

**Figure 1:**
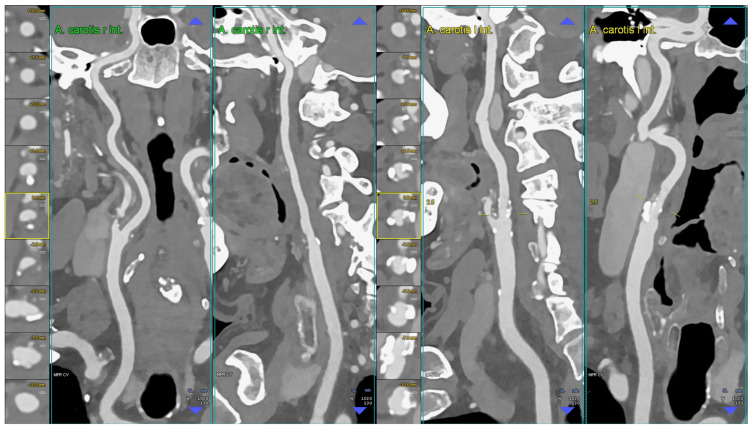
CT angiography of the carotid arteries; moderate bilateral extra-cranial atherosclerotic disease. The figure shows a CT angiography of the carotid arteries performed using a whole body PCCT scanner but using reconstruction parameters comparable to the best ones applicable by a state-of-the-art non-PCCT scanner (in summary: 0.6 mm slice thickness with 0.4 mm slice increment, medium convolution kernel); in particular, the figure shows orthogonal longitudinal multiplanar reconstruction of the right and left carotid artery axes with corresponding series of axial cross-sections of the respective bifurcation on left of each one. The image shows mild predominantly non-calcified atherosclerosis on the right bifurcation and mild predominantly calcified atherosclerosis on the left bifurcation. Abbreviations: CT = computed tomography; PCCT = photon-counting CT.

**Figure 2:**
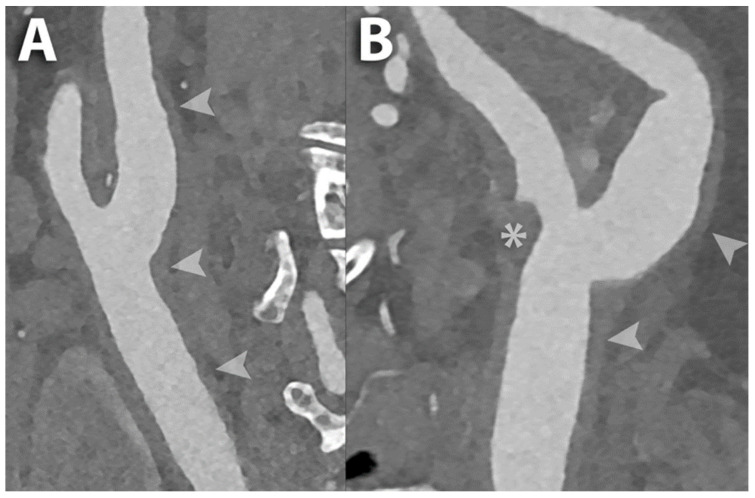
PCCT angiography of the carotid arteries; mild bilateral extra-cranial atherosclerotic disease. The figure shows a magnified view of a PCCT angiography of the carotid arteries in longitudinal multiplanar reconstruction of the right (**A**) and left (**B**) carotid bifurcation. The image shows mild diffuse predominantly non-calcified atherosclerosis bilaterally (arrowheads) with a more pronounced non-calcified plaque at the ostium of the external carotid artery on the left (* in (**B**)). The scan was performed on a commercial whole-body dual-source photon-counting CT scanner (NAEOTOM Alpha, Siemens Healthineers, Erlangen, Germany) with 0.2/0.1 mm slice thickness/increment, FOV 120–140 mm, resolution matrix of 1024 × 1024 voxels on the source axial reconstructions with a kernel filtering of Bv60-72 (vascular kernel medium-sharp/sharp) and with maximum intensity of Quantum Iterative Reconstruction (QIR 4). The displayed image resolution is 100 microns. Abbreviations: CT = computed tomography; PCCT = photon-counting CT; FOV = field of view.

**Figure 3:**
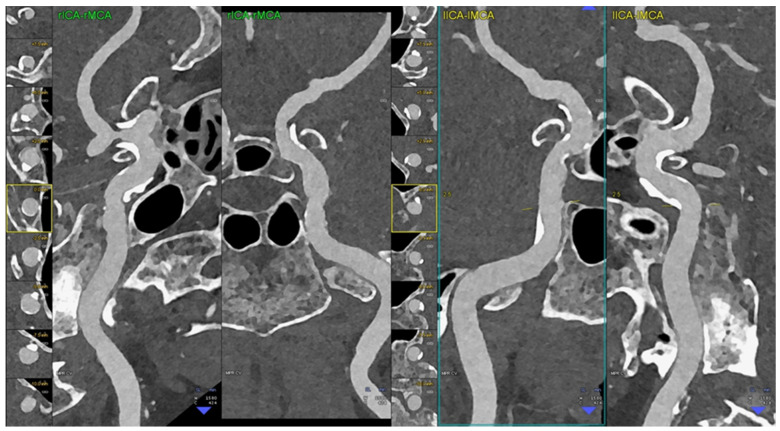
PCCT angiography of the intra-cranial arteries; mild bilateral atherosclerotic disease. The figure shows a magnified view of a PCCT angiography of the intra-cranial arteries in orthogonal longitudinal multiplanar reconstruction of the right and left intra-petrous internal carotid arteries. The image shows mild diffuse predominantly calcified atherosclerosis bilaterally. The scan was performed on a commercial whole-body dual-source photon-counting CT scanner (NAEOTOM Alpha, Siemens Healthineers, Erlangen, Germany) with 0.2/0.1 mm slice thickness/increment, FOV 120 mm, resolution matrix of 1024 × 1024 voxels on the source axial reconstructions with a kernel filtering of Bv60-72 (vascular kernel medium-sharp/sharp) and with maximum intensity of quantum iterative reconstruction (QIR 4). The displayed image resolution is 100 microns. Abbreviations: CT = computed tomography; PCCT = photon-counting CT; FOV = field of view.

**Figure 4:**
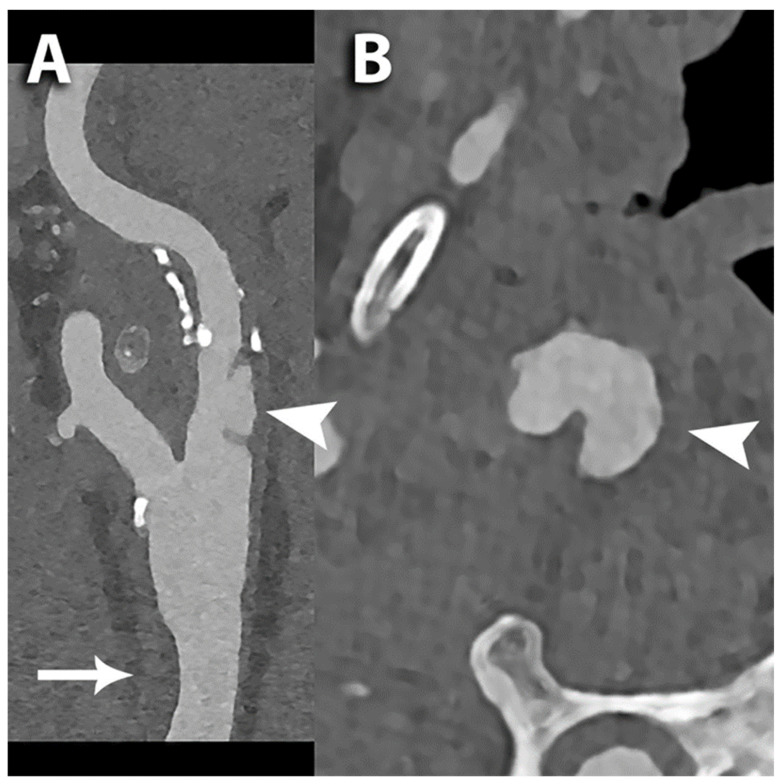
PCCT angiography of the carotid arteries; ulceration of the internal carotid artery. The figure shows a PCCT angiography of the carotid arteries and in particular a longitudinal multiplanar reconstruction (**A**) and an axial cross-sectional image orthogonal to the longitudinal axis of the proximal internal carotid artery (**B**); arrowheads in (**A**,**B**) are indicating a focal deep ulceration of the wall of the ICA; above the ulceration there is mixed plaque along the ICA; the arrow in A shows a moderate focal non-calcified eccentric thickening of the ventral portion of the distal III of the corresponding common carotid artery. The scan was performed on a commercial whole-body dual-source photon-counting CT scanner (NAEOTOM Alpha, Siemens Healthineers, Erlangen, Germany) with 0.2 mm slice thickness, 0.1 mm reconstruction increment, FOV 140–160 mm, resolution matrix of 1024 × 1024 pixels on the source axial reconstructions with a kernel filtering of Bv60-72-80 (vascular kernel sharp/ultra-sharp) and with maximum intensity of quantum iterative reconstruction (QIR 4). The displayed image resolution is 100 microns. Abbreviations: CT = computed tomography; PCCT = photon-counting CT; ICA = internal carotid artery; FOV = field of view.

**Figure 5:**
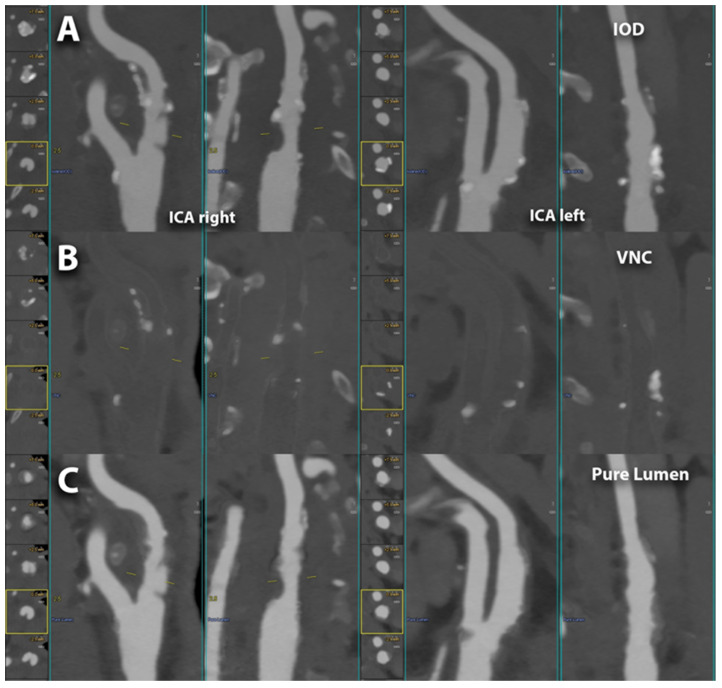
Spectral PCCT angiography of the carotid arteries; spectral capabilities. The figure shows 3 different modalities for the visualization performed with spectral PCCT angiography of the right and left carotid artery bifurcations; the vessels are automatically segmented and displayed in longitudinal orthogonal multiplanar views. The spectral imaging modalities are directly reconstructed as such from the scanner or switchable on the workstation; in the figure, the first row (**A**) represents the IODINE image, the middle row (**B**) represents the virtual non-contrast (VNC) image, and the lower row (**C**) represents the PureLumen image. The scan was performed on a commercial whole-body dual-source photon-counting CT scanner (NAEOTOM Alpha, Siemens Healthineers, Erlangen, Germany) with 0.2/0.4 mm slice thickness, 0.1/0.2 mm reconstruction increment, FOV 140–160 mm, resolution matrix of 512 × 512/1024 × 1024 pixels on the source axial reconstructions with a kernel filtering of Bv48-60 (vascular kernel medium-sharp) and with maximum intensity of Quantum Iterative Reconstruction (QIR 4). Abbreviations: CT = computed tomography; PCCT = photon-counting CT; KeV = Kilo-electron-Volt; FOV = field of view; ICA = internal carotid arteries; IOD = spectral mode with iodine image visualization; VNC = spectral mode with virtual non-contrast (elimination of iodine signal within the images; similar to a pre-contrast scan derived from a contrast enhanced scan); pure lumen = spectral mode with sole visualization luminal content/Iodine (more pure visualization that ignores calcifications as well).

**Figure 6:**
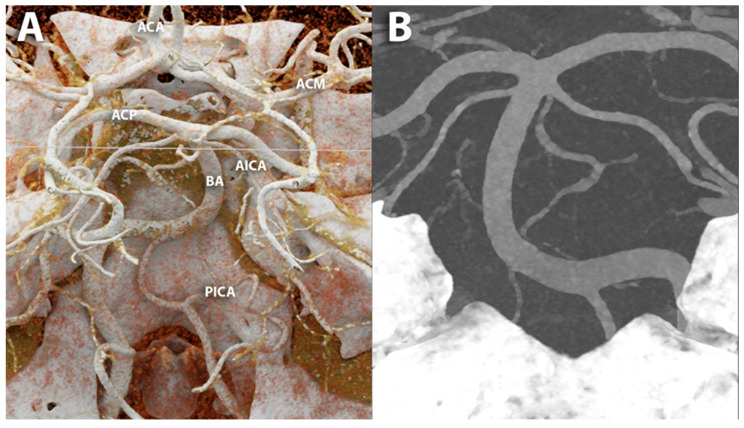
PCCT angiography of the intra-cranial Willis’ circle. The figure shows a PCCT angiography of the brain visualized with cinematic rendering (**A**) and MIP (**B**) focused on the posterior cerebral circulation; highly detailed visualization of smaller branches is easily displayed. The scan was performed on a commercial whole-body dual-source photon-counting CT scanner (NAEOTOM Alpha, Siemens Healthineers, Erlangen, Germany) with 0.2/0.1 mm slice thickness/increment, FOV 120–140 mm, resolution matrix of 1024 × 1024 voxels on the source axial reconstructions with a kernel filtering of Bv60-72 (vascular kernel medium-sharp/sharp) and with maximum intensity of quantum iterative reconstruction (QIR 4). The displayed image resolution is 100 microns. Abbreviations: CT = computed tomography; PCCT = photon-counting CT; ACA = arteria cerebralis anterior; ACM = arteria cerebralis media; ACP = arteria cerebralis posterior; BA = arteria basilaris; AICA = anterior inferior cerebellar artery; PICA = posterior inferior cerebellar artery; FOV = field of view.

This section summarizes the phantom, animal, and clinical studies employing PCCT to evaluate carotid artery disease. Table 1 sums up the impact of the PCCT benefits on the assessment of the carotid artery disease. Clinical case examples are provided to further highlight the potential clinical utility of PCCT in this context (Figure 1, Figure 2, Figure 3, Figure 4, Figure 5 and Figure 6).

### 6.1. Carotid Lumen Evaluation

Several studies have demonstrated the effectiveness of PCCT in improving the assessment of the carotid vessel lumen, owing to its enhanced spatial resolution, soft-tissue contrast, and reduced noise levels.

Li et al. introduced an innovative method for measuring the percent area stenosis based on the material decomposition of dual-energy and multiple-energy CT images [86]. Through computer simulations, the authors demonstrated that the proposed method effectively addressed challenges such as partial volume effects and blooming artifacts. The phantom experiments proved the superiority of the four-threshold PCCT approach over DECT and the two-threshold PCCT strategy in reducing measurement inaccuracies. Furthermore, the implementation of a three-basis-material decomposition on the four-threshold PCCT images enabled to obtain distinct maps delineating the spatial distribution of calcium, iodine, and water, offering valuable insights into the composition and morphology of the vascular structures.

In an ex vivo study (carotid artery specimen), Sartoretti et al. demonstrated the efficacy of PCCT in delineating the carotid vessel lumen [87]. The authors explored the use of an experimental tungsten-based contrast medium to enhance the visualization of carotid vessels, comparing it with conventional iodine-based contrast agents and validating their results against histological specimens as a reference. Their study revealed that employing tungsten as a contrast agent markedly reduced image noise compared to iodine agents and led to improved vessel visualization and vessel wall delineation.

The improved diagnostic accuracy of PCCT in carotid vessel lumen delineation was also demonstrated in in vivo studies. Symons et al. prospectively enrolled sixteen asymptomatic subjects and investigated the image quality of carotid and intra-cranial vessels using PCCT compared to EID-CT [58]. According to two independent radiologists blinded to the detector system, PCCT images were characterized by significantly higher quality scores for both extra-cranial and intra-cranial vessels (all *p* < 0.05), significantly lower subjective image noise (*p* < 0.01), and fewer image artifacts (*p* < 0.001). In their study involving thirty-seven patients, Michael et al. showcased that PCCT delivered exceptional image quality for CT angiography of the head and neck [88]. In particular, the authors reported that polyenergetic reconstructions outperformed monoenergetic reconstructions in terms of both objective (signal, contrast, SNR, and CNR) and subjective image quality parameters.

### 6.2. Carotid Stents

Carotid artery stenting has revolutionized the therapeutic landscape for carotid artery stenosis, offering a less invasive alternative to conventional carotid endarterectomy. This interventional approach has garnered widespread recognition for its efficacy and safety profile, particularly in high-risk patients [89,90]. Despite its widespread adoption, traditional CT systems encounter challenges in accurately assessing carotid stents and surrounding tissues. Metal artifacts, blooming effects, and limitations in spatial resolution often impede the precise evaluation of stent integrity and potential complications post-implantation, such as in-stent restenosis, in-stent thrombosis, and stent fracture or displacement [91,92]. In this context, PCCT offers the potential to overcome the inherent limitations of EID-CT systems, providing clinicians with enhanced diagnostic capabilities and facilitating more effective patient management strategies.

Verelst et al., in their ex vivo phantom study with embedded human-resected and stented arteries, examined the diagnostic accuracy of PCCT in evaluating the appearance of carotid stents compared to conventional EID-CT [93]. Regarding qualitative assessment, stent images acquired with PCCT demonstrated a superior appearance compared to EID-CT (IQR 4–5 vs. IQR 2–3, *p* = 0.010), as determined by two expert readers. Moreover, inter-stent visibility reader scores were notably higher for PCCT images. In terms of quantitative assessment, PCCT achieved more accurate diameter measurements with a reduction in mean error toward the true diameter (0.17 mm ± 0.16 mm vs. 0.59 mm ± 0.26 mm, *p* = 0.001), reduced blooming artifacts (18.3% ± 2.6% vs. 32.4% ± 4.6%, *p* = 0.001), and a higher degree of stent distinction (80.7% ± 7.6% vs. 49% ± 15.8%, *p* = 0.001).

In the proof-of-concept study by Almqvist et al., a patient with a carotid stent was scanned with an innovative silicon-based PCD [94]. Using a high-resolution reconstruction kernel enabled the clear visualization of the individual struts within the stented carotid artery. Moreover, when VMIs at 70 keV were reconstructed using both high-resolution and standard-resolution kernels, signs of intimal hyperplasia were evident within the carotid lumen.

Imaging can provide valuable information for preprocedural planning of carotid interventions, including details about the anatomy and specific configurations of the arch and carotid vessels, as well as tandem lesions, helping to reduce operator-dependent variables, shorten operation time, decrease X-ray exposure, and increase procedural success [95,96,97]. Moreover, it offers insights into the degree and location of calcification and the presence of atherosclerosis, which could influence the risk of post-procedural complications [98,99]. Compared to EID-CT systems, PCCT, thanks to its innovative technology, enables rapid scans with lower radiation doses and reduced contrast administration, as well as minimized artifacts. This offers a unique opportunity for surgeons to visualize the carotid plaque and surrounding structures prior to carotid artery intervention.

### 6.3. Carotid Plaque Evaluation

PCCT, thanks to its improved spatial resolution, contrast-to-noise ratio, reduced artifacts, and enhanced capabilities in material characterization, may enhance the identification of imaging features associated with plaque vulnerability related to cerebrovascular events.

Dahal et al. conducted an ex vivo study to evaluate the PCCT’s capability in quantifying vulnerable plaque features, such as FC thickness, FC area, and LRNC area [100]. They examined 20 excised plaques obtained from carotid endarterectomy, using histopathological measurements as the gold standard. The study revealed no significant differences between the ex vivo spectral PCCT-derived measurements and histopathological measurements of FC thickness, FC area, and LRNC area. The retrospective ex vivo study by Shami et al. investigated which plaque features could be detected with PCCT using carotid endarterectomies from the Carotid Plaque Imaging Project [101]. On PCCT images, density in HU was quantified in different regions of interest corresponding to various plaque components identified by histopathology (calcium, thrombus, lipid core, necrosis, fibrosis, IPH). For each plaque feature, the relationship between HU and energy was analyzed using a mixed-effects model, and a b1 coefficient was extracted. By comparing the b1 coefficients, the authors demonstrated that calcium could be distinguished from all other analyzed plaque features and that there was notable discernibility between other rupture-prone plaque features, particularly between IPH and fibrous cap (*p* = 0.017), lipids (*p* = 0.003), and necrosis (*p* = 0.004). Similarly, thrombus was distinguishable from fibrosis (*p* = 0.048), fibrous cap (*p* = 0.028), lipids (*p* = 0.015), and necrosis (*p* = 0.017).

Few studies investigated the intrinsic spectral capability of PCCT, showcasing its potential to identify key plaque vulnerability features. Healy et al. demonstrated that PCCT could discern several critical characteristics of vulnerable atherosclerotic plaque, including lipid-rich necrotic core, spotty calcification, and plaque ulceration, in surgically obtained specimens from the carotid artery without the administration of contrast media [102]. Si-Mohamed et al. conducted in vitro experiments to distinguish between gold, iodine, and calcium, followed by in vivo experiments involving atherosclerotic rabbits with induced plaques [70]. The authors demonstrated that PCCT, using multiple energy bins, successfully detected gold nanoparticles and differentiated them from calcifications and iodine. Histological analysis validated the uptake of gold nanoparticles by macrophages within plaques, correlating with the concentrations measured in vivo. This association suggests that elevated concentrations of gold nanoparticles detected by PCCT correspond to increased macrophage presence in advanced atherosclerotic plaques.

In a recent case report involving a 63-year-old patient with internal carotid artery dissection and associated pseudoaneurysm who underwent both PCCT and conventional EID-CT, Keser et al. demonstrated that PCCT provided a better identification of the dissection flap, false lumen, and pseudoaneurysm [103].

Another important aspect that should be taken into account in clinical practice is plaque progression, a well-known risk factor for future ischemic events. Longitudinal studies have shown that certain imaging features of plaque composition, including the presence of IPH, significantly increase the risk of plaque progression [104,105]. The enhanced ability of PCCT to assess atherosclerotic plaque composition could offer a valuable tool for non-invasively tracking and monitoring carotid artery plaque progression. Evidence also suggests that lipid-lowering and anti-inflammatory treatments, as well as healthier lifestyle choices, can reduce carotid plaque size and alter its composition, demonstrating plaque regression [106,107]. In this scenario, PCCT represents a valuable imaging modality to evaluate the transition from vulnerable plaques to more stable plaques. Moreover, since PCCT can provide a significant dose reduction compared with conventional CT without compromising image quality, its use may be associated with safer serial monitoring of disease progression and/or regression.

### 6.4. Proliferation of Vasa Vasorum

Vasa vasorum is a network of microvessels located in the artery wall that plays a fundamental role in the pathophysiology of atherosclerosis. Indeed, several studies documented a clear association of increased vasa vasorum density with atherosclerotic plaque growth and progression toward an inflammatory and unstable plaque phenotype prone to rupture and causing clinical events [108,109,110].

In conventional CT systems, the blooming effect poses a significant obstacle to accurately visualizing and quantifying vasa vasorum, particularly given their small size and close proximity to the lumen. Blooming arises from the limited spatial resolution, leading to the spread of contrast from the vessel lumen into neighboring tissues, resulting in signal enhancement.

Marsh et al. developed a forward model using PCCT to mitigate the overestimation of CT numbers near the lumen–wall boundary due to blooming from enhanced arterial lumens [111]. They initially validated their model with a vessel phantom containing both an enhanced lumen and arterial wall. Subsequently, they conducted in vivo vascular perfusion scans on a swine model. Autologous blood injections were administered to induce vasa vasorum proliferation within the arterial wall. The forward model consistently demonstrated reduced blooming contamination within the vessel walls of the affected artery (*p* = 0.0006). The same group evaluated the performance of PCCT in quantifying alterations in blood flow within the arterial wall resulting from the localized expansion of the vasa vasorum [112]. The authors indirectly measured the spatial density of the vasa vasorum in the carotid artery wall by assessing the total contrast enhancement within the wall in a porcine model in which vasa vasorum proliferation was stimulated in the left carotid artery using the right one as a control. By the use of an exact Wilcoxon-signed rank test, it was determined that the enhancement ratio was significantly higher in the injured arteries compared to the control arteries (*p* = 0.013).

## 7. Conclusions

PCCT represents a paradigm shift from conventional CT imaging, propelled by a new generation of X-ray detectors capable of counting individual photons and measuring their energy. The clinical implications of PCCT in carotid artery imaging are manifold. The exceptional spatial resolution, enhanced image quality, and reduced artifacts contribute to more accurate detection and measurement of arterial stenosis. With its ability to finely discriminate between different tissue types, PCCT allows for detailed characterization of plaque morphology and composition, providing clinicians with insights into plaque vulnerability and disease progression.

Large-scale clinical studies are needed to confirm the potential of PCCT to significantly improve diagnostic accuracy, patient outcomes, and the overall management of carotid artery disease by revolutionizing risk stratification strategies and therapeutic interventions.

## Figures and Tables

**Table 1:** Benefits of photon-counting detectors and impact on the assessment of the carotid artery disease.

Benefits of Photon-Counting Detectors	Impact in Carotid Arteries Assessment
Enhanced spatial resolution	Improved assessment of the carotid vessel lumen Improved stent imaging Improved atherosclerotic plaque characterization
Improved contrast and noise	Improved assessment of the carotid vessel lumen Improved stent imaging Dose reduction
Enhanced spectral capabilities	Improved assessment of the carotid vessel lumen Improved atherosclerotic plaque characterization Dose reduction
Reduced artifacts	Improved assessment of the carotid vessel lumen Improved stent imaging Improved atherosclerotic plaque characterization

## Data Availability

Not applicable.
